# Relative effectiveness of bivalent COVID-19 vaccine: a systematic review and meta-analysis

**DOI:** 10.3389/fmed.2023.1322396

**Published:** 2024-02-07

**Authors:** Meng-qun Cheng, Rong Li, Zhi-ying Weng, Gao Song

**Affiliations:** ^1^Department of Reproductive Medicine, The Puer People's Hospital, Pu’er, China; ^2^Department of Pharmacy, The Puer People's Hospital, Pu’er, China; ^3^School of Pharmaceutical Science and Yunnan Key Laboratory of Pharmacology for Natural Products, Kunming Medical University, Kunming, China

**Keywords:** COVID-19, bivalent vaccine, effectiveness, booster immunization, omicron variant

## Abstract

**Objective:**

The rapid development of COVID-19 bivalent vaccines (BVs) has encompassed both the original virus strains and the variant strain. However, the effectiveness of BVs is largely unknown. Therefore, we conducted a systematic review and meta-analysis of the effectiveness of BVs.

**Methods:**

Literature research was conducted through PubMed, Cochrane Library, Embase, and Web of Science up until November 4, 2023. Both randomized control trials and observational studies were considered for inclusion. Pooled estimates were calculated using a random effects model. The Newcastle-Ottawa Scale (NOS) was used to assess the risk of bias in cohort and case–control studies.

**Results:**

A total of 1,174 articles were reviewed and 22 eligible studies were included. All included studies were observational (15 cohort studies, 7 case–control studies). The total number of participants was 39,673,160, and the number of people vaccinated with BVs as an intervention group was 11,585,182. Two mRNA BVs were mainly involved, including the ancestral strain and the BA.1 or BA.4–5 variants. Meta-analysis results showed, compared with the monovalent vaccines (MVs), the relative effectiveness (rVE) of the BVs in COVID-19-associated infections/symptomatic infections, illnesses, hospitalizations, and deaths was 30.90% [95% confidence interval (CI), 8.43–53.37], 39.83% (95% CI, 27.34–52.32), 59.70% (95% CI, 44.08–75.32), and 72.23% (95% CI, 62.08–82.38), respectively. For those aged 50 years and older, BVs provided an additional 49.69% (95% CI, 41.44–57.94) effective protection compared with MVs. During the dominance period of the omicron XBB variant strain, BVs provided an additional 47.63% (95% CI, 27.45–67.82) effective protection compared with MVs.

**Conclusion:**

Our findings show that the rVE of BVs in preventing COVID-19-associated infections, symptomatic infections, illnesses, hospitalizations, and deaths is higher compared to MVs. Particularly for people over 50 years of age and during the Omicron variant XBB dominance phase, BVs provided superior protection. Therefore, BVs may have a broader application in the prevention and control of coronaviruses variant.

## 1 Introduction

In early 2020, the original strain of acute respiratory syndrome coronavirus 2 (SARS-CoV-2) was isolated and sequenced (1, 2). Similar to other viruses, SARS-CoV-2 undergoes evolutionary changes over time (3, 4). Global data indicates the existence of over 1,000 variants of the novel coronavirus (5). Some of these variants have gained significant attention due to their rapid population spread and clinical significance. Currently, there are five novel coronavirus variants that are classified by WHO as variants of concern (VOCs), namely Alpha, Beta, Gamma, Delta and Omicron (6–8).

The Omicron variant (B.1.1.529) and its sub-lineages were first reported in Botswana, followed by South Africa in January 2021 (9, 10). Over time, the Omicron sub-lineage has exhibited increasing replication advantage, ultimately replacing the previous four notable variants of the novel coronavirus. Omicron was first reported in South Africa at the end of November 2021, and was subsequently replaced by the BA.2, BA.4, BA.5 and BA.13 sublines. Additional sub-lineages of the Omicron variant include BQ.11, BQ.7, BF.2, BA.75.1, XBB, XBB.1, and XBB.5.4. These sub-lineages have evolved from various previously circulating sub-lineages, contributing to the escalating infection rates worldwide.

In the realm of vaccine development targeting the spike protein of the original SARS-CoV-2 virus, monovalent vaccines (MVs) stand out, such as the mRNA vaccines manufactured by Pfizer-BioNTech and Moderna, vector vaccines produced by Johnson & Johnson and AstraZeneca, as well as subunit protein vaccines created by Novavax. The initial Corona Virus Disease 2019 (COVID-19) vaccine was effective and helped reduce symptomatic emergency department visits, hospitalizations, ventilator use, and mortality (11–13). In a phase 2 study of an mRNA vaccine, clinical effectiveness was as high as 95% (14). However, recent research has revealed that MVs exhibit significantly weaker neutralizing effects against the Omicron variant compared to the original strain or other variants (15, 16). Furthermore, numerous monoclonal antibody preparations developed thus far have proven ineffective against this variant (17, 18). To tackle this new challenge, bivalent vaccines (BVs) have emerged, offering the ability to encode the spike protein of both the original SARS-CoV-2 strain and its variant strains. COVID-19 mRNA BVs, such as Omicron BA.1 or BA.4–5 variants, developed using mRNA technology, are currently being investigated in some countries.

The rapid spread of Omicron variants to all countries has accelerated the emergency use authorization for Omicron variants by BVs. In this study, our aim was to address the uncertainty surrounding the effectiveness of BVs by conducting a comprehensive systematic review and meta-analysis. Through our study, we have filled this important knowledge gap by systematically evaluating the relative effectiveness (rVE) of BVs compared to MVs and meta-analyzing clinical outcome indicators.

## 2 Methods

This systematic review was completed following the guidelines in the Preferred Reporting Items for Systematic Evaluation and Meta-Analysis (PRISMA) (19).

### 2.1 Inclusion and exclusion criteria

Literature inclusion criteria included: (1) the intervention group was bivalent COVID-19 vaccinated, while the control group consisted primarily of study subjects vaccinated with monovalent COVID-19 vaccine; (2) the type of trial was a randomized controlled trial (RCT) or observational study (e.g., cohort or case–control study); and (3) Report at least one vaccine’s effectiveness result of interest:COVID-19-associated infection, symptomatic infection, hospitalization, emergency department/urgent care (ED/UC) visit, serious illness, or death. Exclusion criteria included: (1) studies that were not peer-reviewed; (2) study protocols, reviews, commentaries, news, case reports, conference abstracts, animal studies, *in vitro* experiments, and antibody-neutralization analyses; and (3) unavailability of the full text.

### 2.2 Search strategy

Literature research was conducted through PubMed, Cochrane Library, Embase, and Web of Science up until November 4, 2023. A comprehensive search was performed using Boolean logic and a “subject + free word” approach. The main English search terms included: “COVID-19,” “SARS-CoV-2,” “vaccine,” and “bivalent.” Please refer to the attached table for specific search strategies (Supplementary Appendix S1).

### 2.3 Study selection process

The study was independently assessed by two researchers (CMQ and LR) based on the aforementioned criteria, and any discrepancies were resolved by a third researcher (SG or WZY). All search results obtained from the database were imported into Endnote X9 software, and duplicate literature was eliminated using the software’s deduplication function. Initial screening involved reviewing the titles and abstracts, and secondary screening involved reviewing the full text while documenting the screening outcomes and reasons.

### 2.4 Data extraction and collection

Two researchers (CMQ and LR) independently extracted data based on a predetermined form in Microsoft Excel. (1) Basic study information such as the first author, publication year, type of BVs; (2) details regarding the study, including study design, age range, study period, population characteristics, study location, number of vaccine doses administered, prevalent strains, and duration of follow-up; and (3) effectiveness findings of the COVID-19 vaccine. In the effectiveness data extraction process, we prioritize the inclusion of aggregated effectiveness data. If aggregated data were not available, data from subgroups were included in detail.

### 2.5 Outcomes of interest

The primary clinical outcome indicator is the rVE of BVs compared to MVs in preventing COVID-19-associated infections, symptomatic infections, hospitalizations, illness and deaths.

Secondary indicator, rVE of BVs compared to MVs in populations >50 years of age or older and during periods of omicron subline XBB dominance.

### 2.6 Quality assessment

Two researchers (CMQ and LR) independently assessed the risk of bias in cohort and case–control studies using the Newcastle-Ottawa Scale (NOS) (20). The NOS consists of eight categories related to methodological quality, with a maximum score of nine. A total score of 7–9 was considered a high-quality study, 4–6 a moderate-quality study, and 1–3 a low-quality study. Two researchers reviewed the studies and judged the risk of bias, and disagreements, if any, were resolved jointly by a third researcher (SG or WZY).

### 2.7 Statistical analyses

All analyses were visualized using Stata 17 statistical software. Pooled estimates were calculated using the DerSimonian and Laird method for a random effects model with a 95% confidence interval (CI) (21). Where studies did not report pooled data or where multiple subgroups of categorical data existed, we selected data with lower rVE for meta-analysis to avoid amplified effects. Statistical heterogeneity of the results was using the I^2^ statistic. An I^2^ statistic value greater than 50% was considered indicative for substantial heterogeneity (22). Q-tests were used for subgroup comparisons, variables between subgroups were considered significant when the value of p for subgroup differences was less 0.05. The publication bias was studied by visual inspection of the funnel plot symmetry as well as by Egger’s test (asymmetry considered if *p* < 0.05) (23). Sensitivity analysis was performed for outcomes that included more than 10 studies. *p* < 0.05 were considered statistically significant differences.

## 3 Results

### 3.1 Literature search

**Figure 1:**
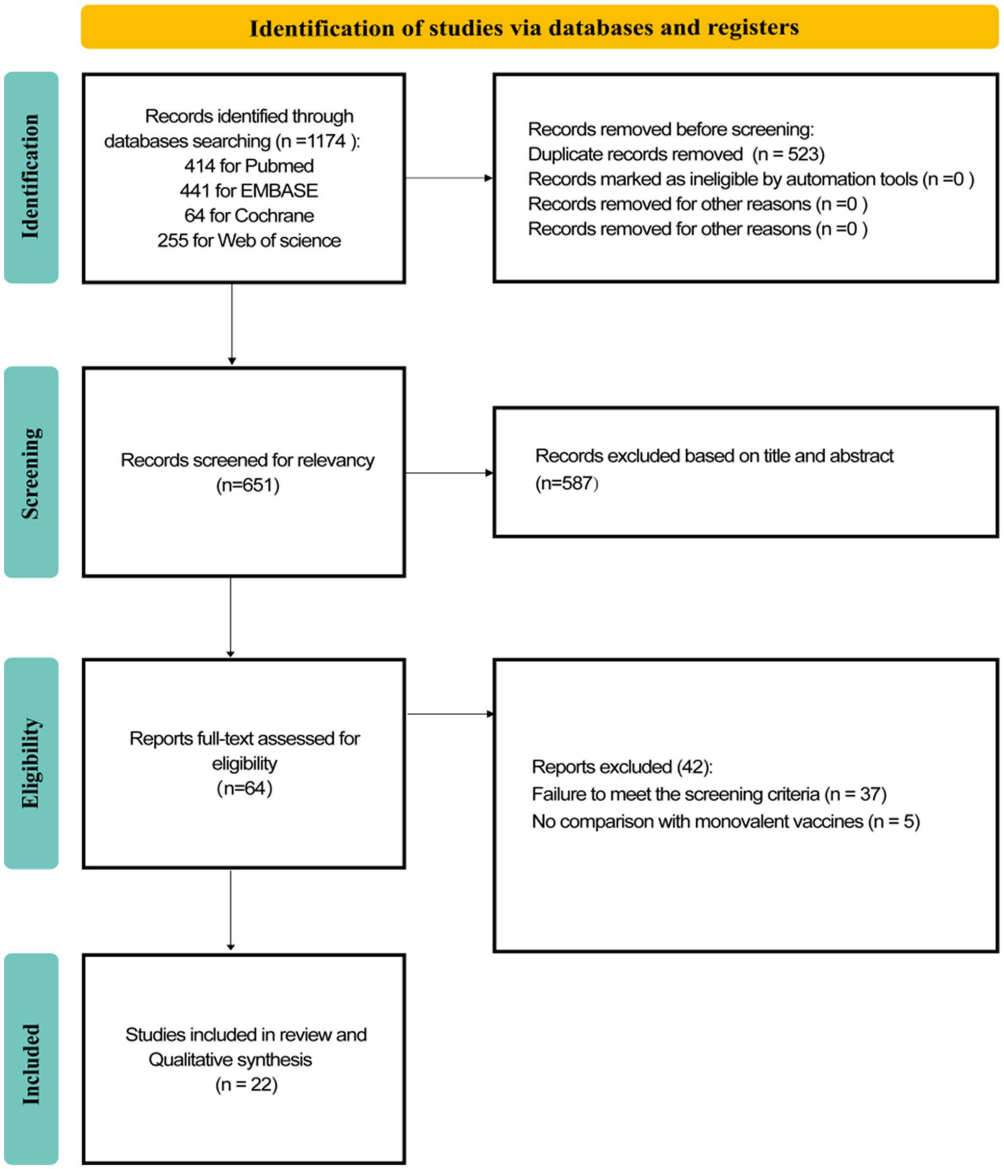
Flow chart of literature screening.

A total of 1,174 records were retrieved from these four databases. Among them, 414 were from PubMed, 441 from EMBASE, 64 from the Cochrane Library, and 255 from the Web of Science database. After removing 523 duplicate records, the titles and abstracts of the remaining articles were screened. We excluded 587 reviews and other studies that did not meet the exclusion criteria. When we read the full text of the remaining 64 studies, 42 were excluded (Figure 1), resulting in 22 studies (23–45) that were included and extracted for this systematic review. All included studies were observational, consisting of 15 cohort studies and 7 case–control studies. No RCTs were identified. The detailed literature screening process is presented in Figure 1.

### 3.2 Evaluation of methodological quality of included studies

As shown in Table S1, the majority of all the studies we included were assessed as having moderate and high methodological quality. Specifically, 5 of the 15 cohort studies were rated as moderate and 10 as high, and 1 of the 7 case–control studies was rated as low, 3 as moderate, and 3 as high.

### 3.3 Basic characteristics of the included studies

Of the 22 included studies, 12 involved only mRNA BVs (including BA.4–5), 2 involved only mRNA BVs (including BA.1), 7 involved mixed mRNA BVs (including BA.4–5 or BA.1), and the remaining 1 study did not specify the specific type of BVs. The total number of participants was 39,673,160, and the number of people vaccinated with BVs as an intervention group was 1,158,5,182. The majority of study sites were located in the United States, followed by Italy and Japan. All BVs were booster doses, while most of the MVs were booster doses. The prevalent variants during the study period included sublines of Omicron strains BA.2, BA.4, BA.5, and XBB. The baseline characteristics of the participants are presented in Table S2.

### 3.4 rVE of BVs for COVID-19-associated infections or symptomatic infections

**Figure 2:**
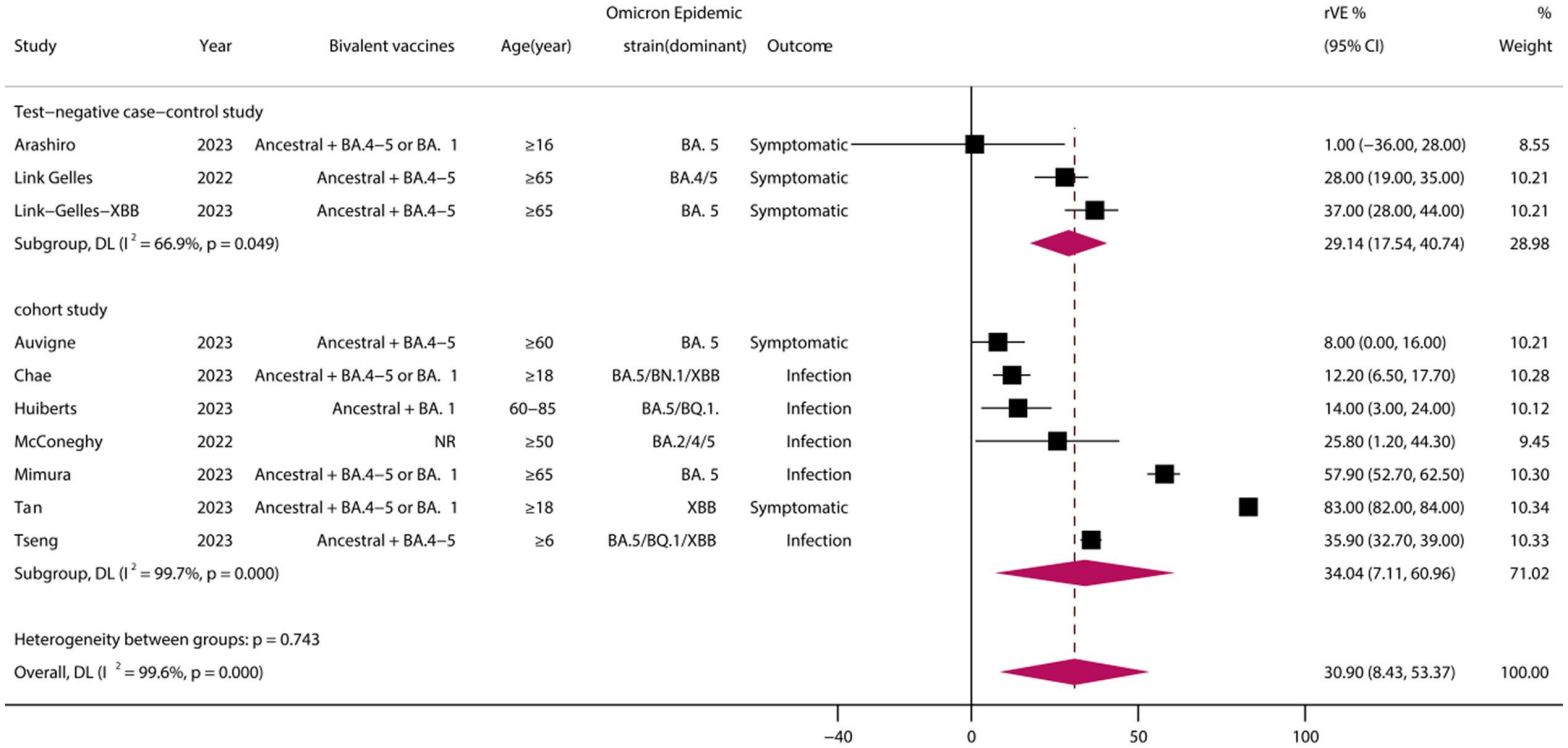
Relative effectiveness of bivalent vaccines in COVID-19-associated infections or symptomatic infections. rVE, relative effectiveness; CI, confidence interval.

A total of 10 eligible studies were included (23, 27, 28, 31, 34, 35, 37, 38, 42, 45), comprising 7 cohort studies and 3 test-negative case–control studies. Meta-analysis demonstrated that vaccination with BVs could enhance the effectiveness of COVID-19-associated infection or symptomatic infection by at least approximately 30.90% (95% CI, 8.43–53.37) compared to MVs. However, heterogeneity was high (*I*^2^ = 99.6%) (Figure 2). Subgroup analysis of study types revealed no significant differences between cohort and case–control studies (*p* = 0.743). Funnel plots and Egger’s test indicated no publication bias in the meta-analysis of the rVE of BVs (test: *t* = −1.65, df = 12, *p* = 0.137). Adopting the trim-and-fill method, the adjusted rVE (36.94, 95% CI, 16.95–56.94) closely aligned with the original results (Supplementary Figure S1).

### 3.5 rVE of BVs for COVID-19-associated illness

**Figure 3:**
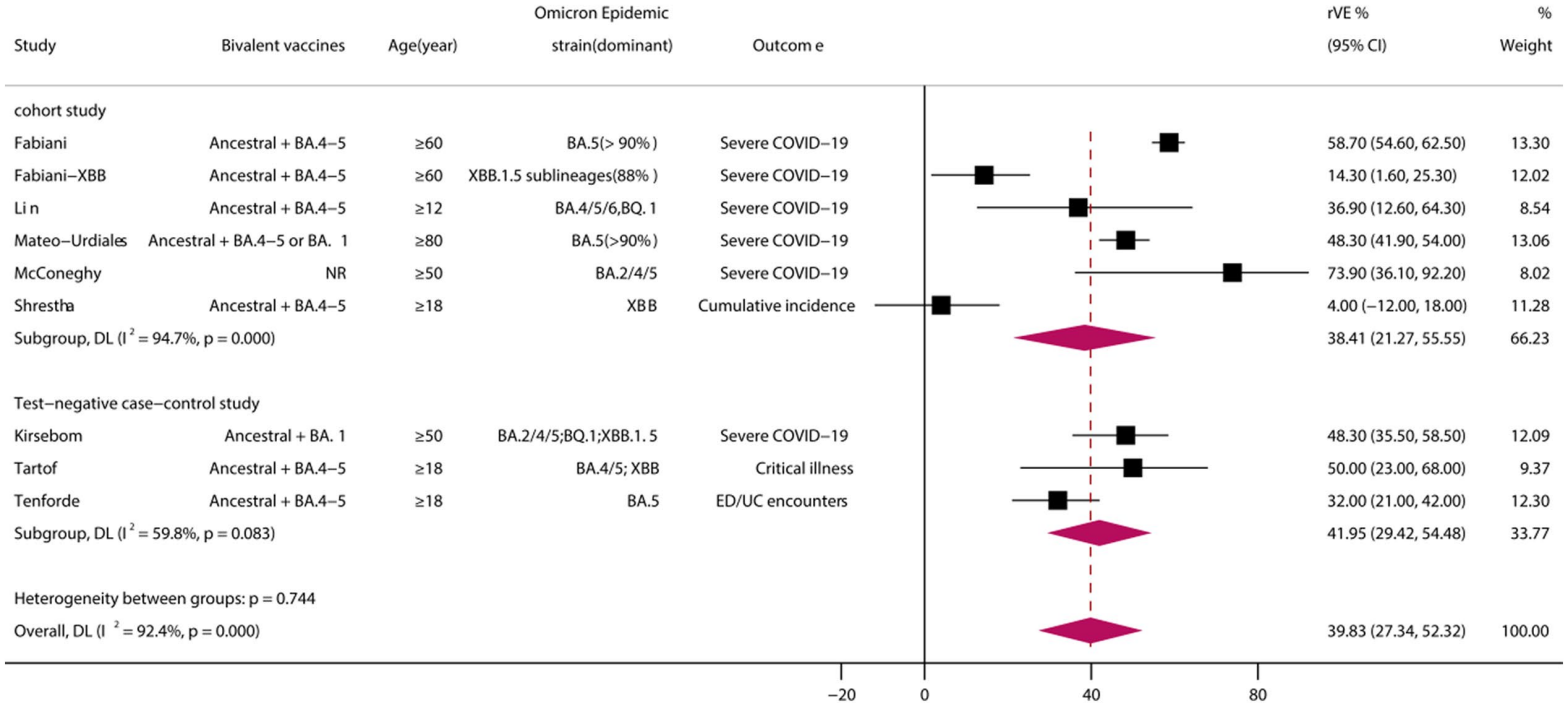
Relative effectiveness of bivalent vaccines in COVID-19-associated illness. rVE, relative effectiveness; CI, confidence interval.

A total of 9 eligible studies were included (29, 30, 32, 33, 36, 37, 39, 43, 44), comprising 6 cohort studies and 3 test-negative case–control studies. Meta-analysis demonstrated that vaccination with BVs could enhance the effectiveness of COVID-19-associated illness by at least 39.83% (95% CI, 27.34–52.32) when compared to MVs (Figure 3). However, heterogeneity was high (*I*^2^ = 92.4%). Subgroup analysis of study types showed no significant differences between cohort and case–control studies (*p* = 0.744).

### 3.6 rVE of BVs for COVID-19-associated hospital admission

**Figure 4:**
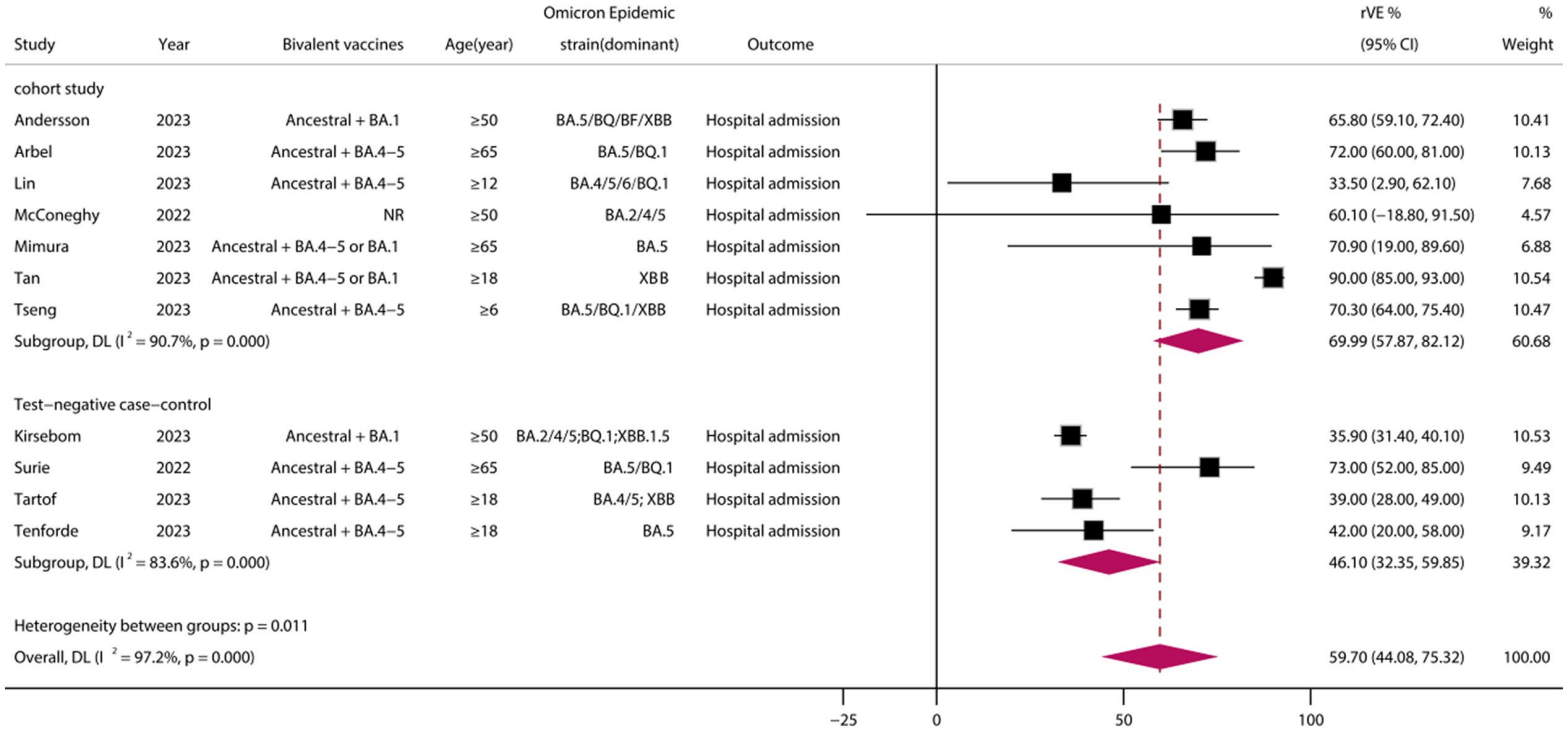
Relative effectiveness of bivalent vaccines in COVID-19-associated hospital admission. rVE, relative effectiveness; CI, confidence interval.

A total of 11 eligible studies were included (24, 26, 32, 33, 37, 38, 41–45), comprising 7 cohort studies and 4 test-negative case–control studies. Meta-analysis demonstrated that vaccination with BVs could enhance the effectiveness of COVID-19-associated hospital admission by at least 59.70% (95% CI, 44.08–75.32) when compared to MVs (Figure 4). However, heterogeneity was high (*I*^2^ = 97.2%). Subgroup analysis of study types showed a significant difference between cohort and case–control studies (*p* = 0.011), with the rVE of the BVs being lower in case–control studies than in cohort studies (46.00 vs. 69.99, *p* = 0.011). Funnel plots and Egger’s test indicated no publication bias in the meta-analysis of the rVE of BVs (test: t = −0.36, df = 11, *p* = 0.718) (Supplementary Figure S1).

### 3.7 rVE of BVs for COVID-19-associated death

**Figure 5:**
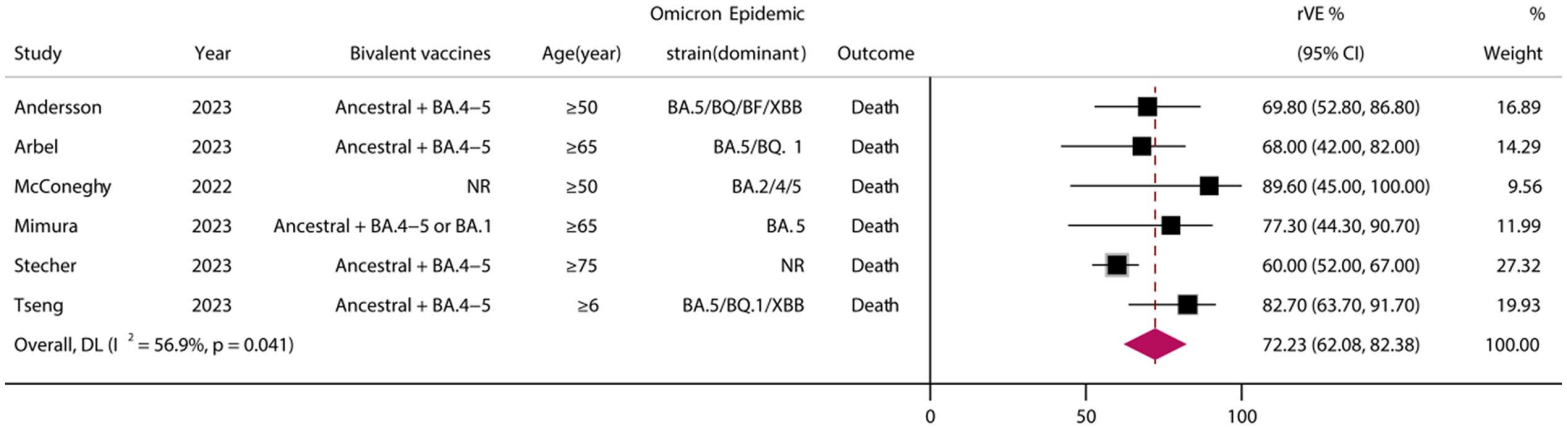
Relative effectiveness of bivalent vaccines in COVID-19-associated death. rVE, relative effectiveness; CI, confidence interval.

A total of six eligible studies were included (24, 26, 37, 38, 40, 45), all of which were cohort studies. Meta-analysis demonstrated that vaccination with BVs could enhance the effectiveness of COVID-19-associated death by at least 72.23% (95% CI, 62.08–82.38) when compared to MVs (Figure 5).

### 3.8 rVE of BVs at ≥50 years of age and in the XBB dominant stage

**Figure 6:**
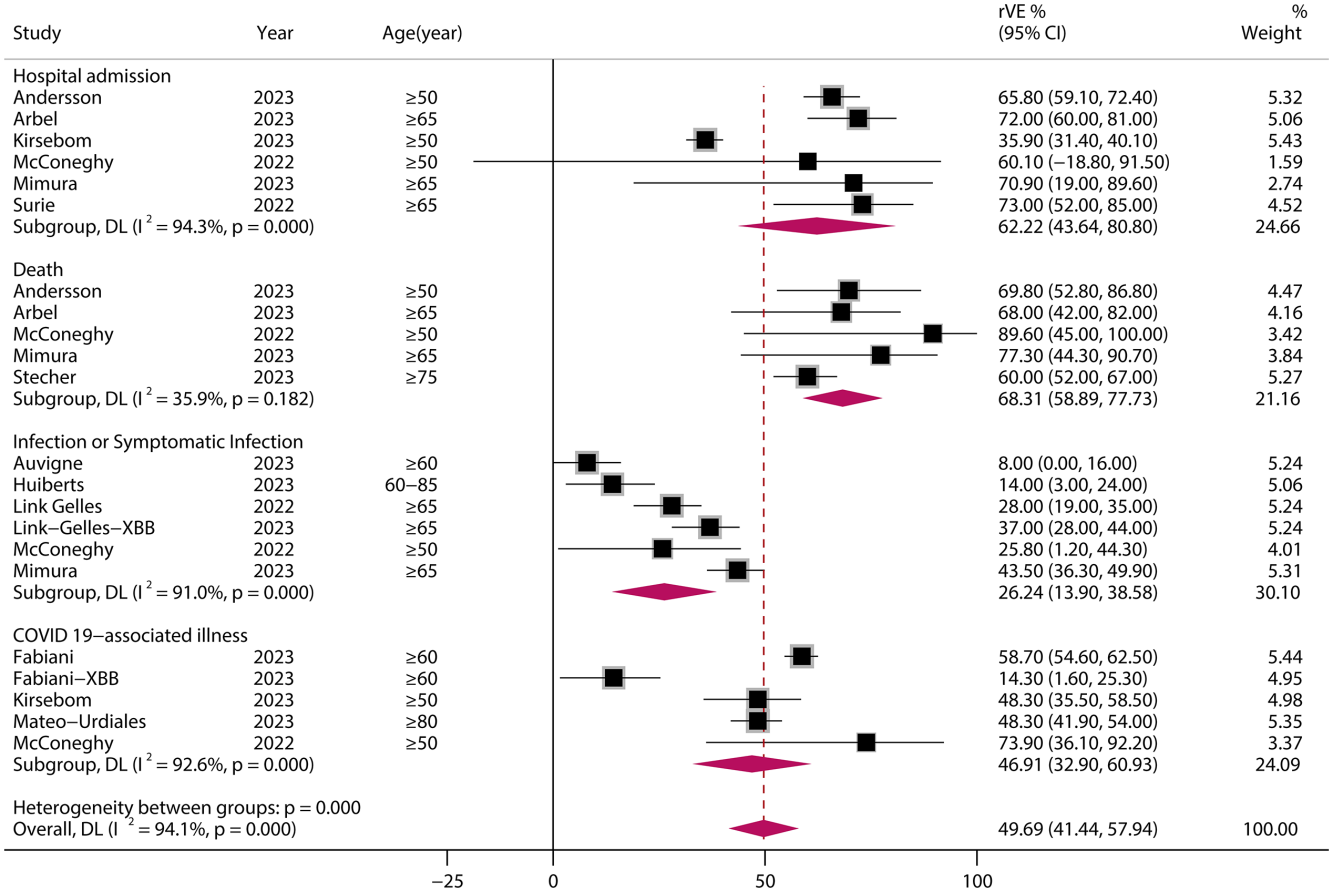
Relative effectiveness of bivalent vaccine in people aged ≥50 years. rVE, relative effectiveness; CI, confidence interval.

**Figure 7:**
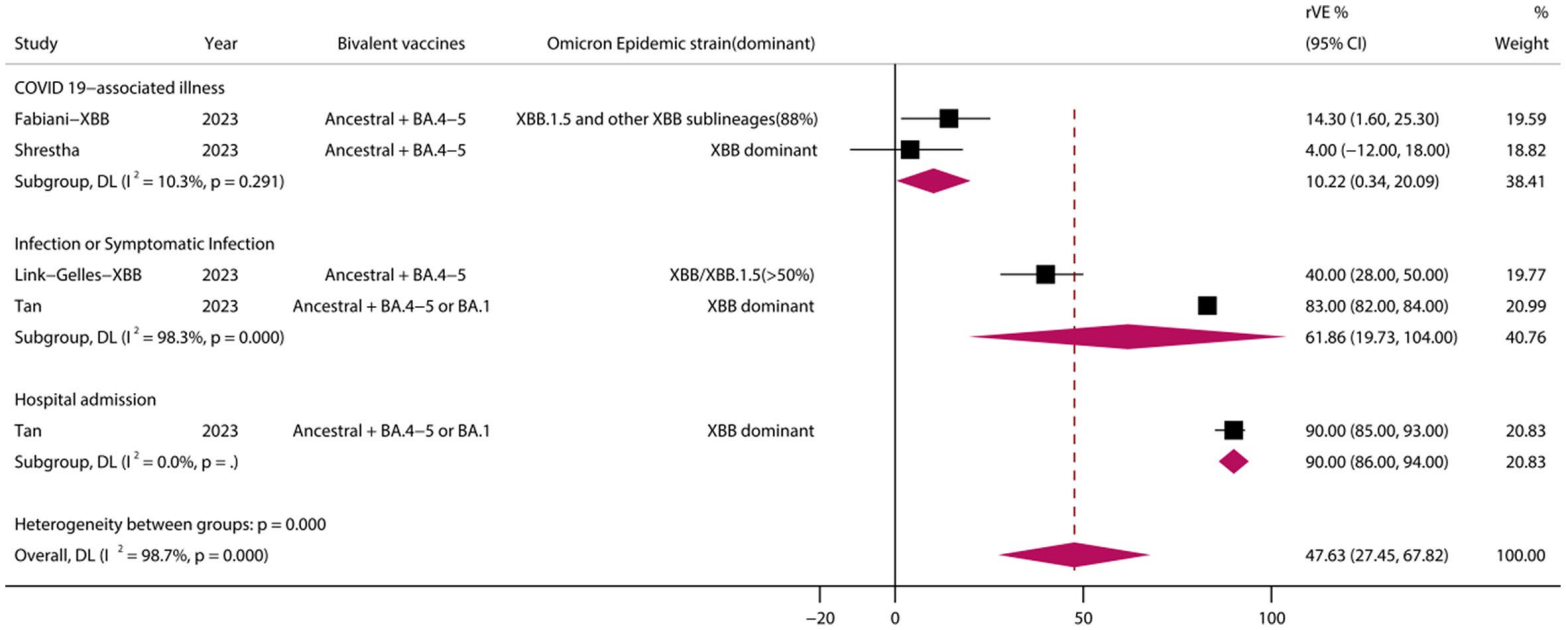
Relative effectiveness of bivalent vaccine during the dominance phase of the omicron XBB mutant strain. rVE, relative effectiveness; CI, confidence interval.

A analysis of those aged >50 years showed that the rVE of BVs compared with MVs was 49.69% (95% CI, 41.44–57.94) (Figure 6). The rVE of the BVs compared with the MVs was 47.63% (95% CI, 27.45–67.82) during the period of epidemiologic predominance of the omicron XBB variant (Figure 7).

### 3.9 Sensitivity analysis

To explore the stability of the results of the meta-analysis, we performed sensitivity analyses on studies with a number of included studies greater than 10 by excluding each study individually. The results showed no significant reduction in the rVE of BVs (Supplementary Figure S2).

## 4 Discussion

The results of our meta-analysis demonstrated that BVs were more effective than MVs across various clinical outcome indicators related to COVID-19. Specifically, the rVE of BVs for COVID-19-associated infections/symptomatic infections, illness, hospitalization, and death was estimated to be 30.90% (95% CI, 8.43–53.37), 39.83% (95% CI, 27.34–52.32), 59.70% (95% CI, 44.08–75.32), and 72.23% (95% CI, 62.08–82.38), respectively. These findings highlight the significant superiority of BVs in terms of effectiveness across multiple clinical outcomes and underscore the importance of considering BVs as a preferred intervention strategy in the Omicron era.

Although antiviral drugs (e.g., Paxlovid) are widely available, they are currently not a substitute for effective vaccination (46). Because of their low economic cost, wide applicability, and safety, vaccines remain a key tool in the fight against novel coronaviruses (47). To reconstruct the target virus, biomedical scientists tracked the functional and structural evolution of SARS-CoV-2 for efficient vaccine development (48). Two mRNA vaccines have been developed that were prepared by nanoparticle encapsulation, nuclear degradation, and encoding SARS-CoV-2 spiking proteins, BNT162b2 (49) and mRNA-1273 (50). Clinical trials validated the efficacy of these two mRNA vaccines with more than 90% protection against COVID-19 and a favorable safety profile. However, SARS-CoV-2 viruses may modulate protective immune responses by developing immune evasion mechanisms to provide a more stable ecological niche (51, 52). In the face of the emergence of the Omicron mutant strain, monovalent vaccines encoding only the original strain do not appear to be able to establish an effective immune barrier, as immune escape leads to reduced vaccine effectiveness. This may be related to the fact that the SARS-CoV-2 genome may encode two additional accessory proteins, such as ORF9c and ORF10, which play key roles in viral replication and immune escape (53). Therefore, the development of more advanced specific vaccines is of particular importance, and bivalent vaccines have followed.

The discussion on the evolution of vaccination strategies continues. A meta-analysis covering eight studies showed that BVs were similar in inducing immunogenicity against ancestral strains compared to MVs. However, the findings also showed that the BVs increased immunogenicity against other mutant strains by an additional approximately 33–50%. The study states that if one chooses to receive booster vaccinations, the BVs should be preferred as a booster based on the current study. However, it has also been argued that continually updating booster vaccines in response to each new dominant strain may not be as beneficial, especially for younger people (54). The results of our Meta-analysis show that for older adults, the BVs retains its ability to reduce the risk of hospitalization and death. These findings emphasize the critical role of booster bivalent vaccination. Therefore, it is becoming increasingly important to develop more effective vaccination strategies, especially for high-risk populations.

SARS-CoV-2 continue to evolve with increasing immune escape, and ongoing surveillance and evaluation are critical. Among other things, the emergence of a new mutant of SARS-CoV-2, XBB.1.5, poses a new challenge to the protection of COVID-19 vaccines. On January 21, 2023, the Centers for Disease Control and Prevention (CDC) updated data on the detection of SARS-CoV-2 mutant strains in the United States, which showed a rise in the proportion of XBB.1.5–49.1%. Mutants in the XBB sub-lineage have been found to become dominant mutants. In light of this, an assessment of the effectiveness of vaccine protection against the XBB.1.5 mutant and its impact is particularly important. on January 25, 2023, the CDC published a highly publicized study of vaccine efficacy of a BVs against the SARS-CoV-2 XBB/XBB.1.5 in a real-world setting. To this point, our meta-analysis of studies related to the period of XBB dominance showed that BVs still provided effective additional protection against COVID-19-associated infections and hospitalizations compared with MVs, with a pooled rVE of 47.63% (95% CI, 27.45–67.82). Vaccination with BVs may be a superior option in the XBB era.

The main limitation of this study is the heterogeneity and incompleteness of the data collected. To avoid amplified effects, we followed the principle of least difference in selecting data for meta-analysis. We attempted to perform subgroup analyses to explore the causes of heterogeneity, but incomplete data prevented a comprehensive subgroup analysis. In addition, there are fewer studies on the dominance period of Omicron XBB variants, and thus interpretations need to be cautious. Moreover, All of the included studies were observational. Although most of these observational studies were of moderate or high quality according to the NOS, this scale itself has some limitations. Also, because they are observational studies, all studies (even the high quality ones) inevitably have some risk of bias. However, despite the limitations of existing real-world studies, the expected validity of BVs is in line with what would be expected in the current context based on the results of this systematic review and meta-analysis.

## 5 Conclusion

Our findings show that the rVE of BVs in preventing COVID-19-associated infections, symptomatic infections, illnesses, hospitalizations, and deaths is higher compared to MVs. Particularly for people over 50 years of age and during the Omicron variant XBB dominance phase, BVs provided superior protection. Therefore, BVs may have a broader application in the prevention and control of coronaviruses variant.

## Data availability statement

The original contributions presented in the study are included in the article/Supplementary material, further inquiries can be directed to the corresponding authors.

## Author contributions

M-qC: Conceptualization, Data curation, Investigation, Methodology, Software, Supervision, Validation, Visualization, Writing – original draft, Writing – review & editing. RL: Data curation, Formal analysis, Methodology, Validation, Visualization, Writing – review & editing. GS: Conceptualization, Data curation, Formal analysis, Funding acquisition, Investigation, Methodology, Project administration, Resources, Software, Supervision, Validation, Visualization, Writing – original draft, Writing – review & editing. Z-yW: Conceptualization, Funding acquisition, Project administration, Writing – review & editing.
