# Supplementary material for: Relative effectiveness of bivalent COVID-19 vaccine: a systematic review and meta-analysis
Source: Front Med (Lausanne). 2024 Feb 7;10:1322396. doi: 10.3389/fmed.2023.1322396 (PMC10879625; doi:10.3389/fmed.2023.1322396)
Supplement: Supplementary file 1 [file Data_Sheet_1.doc]

**Supplementary Material**

**Appendix S1. Literature search strategy**

**Coverage: from the inception to November 4, 2023**

| **Database** | **Result** | **Search strings** |
| --- | --- | --- |
| PubMed | **414** | #1 COVID-19[Mesh] OR SARS-CoV-2[Mesh] OR "COVID-19 Vaccines"[Mesh] OR COVID-19[Title/Abstract] OR SARS-CoV-2[Title/Abstract]  #2 vaccines[Mesh] OR vaccination[Mesh] OR immunization[Mesh] OR vaccin*[Title/Abstract] OR immuniz*[Title/Abstract] OR booster[Title/Abstract] OR Efficacy[Title/Abstract] OR effectiveness[Title/Abstract]  #3 "vaccines, combined"[MeSH Terms] OR ("bivalent"[All Fields] AND "vaccines"[All Fields]) OR "bivalent vaccines"[All Fields]  #1 AND #2 AND #3 |
| Cochrane Central Register of Controlled Trials | **64** | #1 MeSH descriptor: [COVID-19] explode all trees  #2 MeSH descriptor: [SARS-CoV-2] explode all trees  #3 #1 or #2  #4 ("COVID-19" or "COVID 19" or "SARS-CoV-2" or "SARS Coronavirus 2" or "Severe Acute Respiratory Syndrome Coronavirus 2" or "2019-nCoV" or "Coronavirus Disease-19" or "Coronavirus Disease 19" or "Coronavirus Disease 2019" or "Coronavirus Disease-2019 Virus" or nCoV or "Novel Coronavirus"):ti,ab,kw (Word variations have been searched)  **#5 #3 or #4**  #6 MeSH descriptor: [COVID-19 Vaccines] explode all trees  #7 MeSH descriptor: [Vaccines] in all MeSH products  #8 #6 or #7  #9 (vaccin* or immunis* or immuniz* or inoculation or booster):ti,ab,kw or "efficacy" OR "effectiveness"(Word variations have been searched)  **#10 #8 or #9**  #11 ("bivalent") (Word variations have been searched)  **#12 #5 AND #10 AND #11** |
| Web of Science | **255** | (TS=("COVID-19" OR "COVID 19" OR "SARS-CoV-2" OR "SARS Coronavirus 2" OR "Severe Acute Respiratory Syndrome Coronavirus 2" OR "2019-nCoV" OR "2019 nCoV" OR "nCoV-19" OR "nCoV 19" OR "Coronavirus Disease-19" OR "Coronavirus Disease 19" OR "Coronavirus Disease 2019" OR "Coronavirus Disease-2019 Virus" OR "nCoV" OR "Novel Coronavirus") **AND** (TS="vaccin*" OR "vaccination" OR "immunization" OR "inoculation" OR "booster" OR "Efficacy" OR "effectiveness") **AND** (TS=("bivalent"))  Indexes=SCI-EXPANDED, SSCI, A&HCI, CPCI-S, CPCI-SSH, BKCI-S, BKCI-SSH, ESCI, CCR-EXPANDED, IC Timespan = All years |
| EMBASE | **441** | ('coronavirus disease 2019'/exp OR 'severe acute respiratory syndrome coronavirus 2'/exp OR 'covid 19':ab,ti OR 'sars-cov-2':ab,ti OR 'sars coronavirus 2':ab,ti OR 'severe acute respiratory syndrome coronavirus 2':ab,ti OR '2019-ncov':ab,ti OR '2019 ncov':ab,ti OR 'ncov-19':ab,ti OR 'ncov 19':ab,ti OR 'coronavirus disease-19':ab,ti OR 'coronavirus disease 19':ab,ti OR 'coronavirus disease 2019':ab,ti OR 'coronavirus disease-2019 virus':ab,ti OR 'ncov':ab,ti OR 'novel coronavirus':ab,ti) AND ('vaccine'/exp OR 'sars-cov-2 vaccine'/exp OR 'vaccination'/exp OR 'immunization'/exp OR 'vaccin*':ab,ti OR 'immunis*':ab,ti OR 'immuniz*':ab,ti OR 'booster':ab,ti OR 'efficacy' OR 'effectiveness') AND ('bivalent') |

**Table S1 Methodological quality assessment of the included studies**

| Study | Study design | Study population/case selection | | | | Comparability  Item 5 | Exposure/Results | | | NOS | Evaluation quality |
| --- | --- | --- | --- | --- | --- | --- | --- | --- | --- | --- | --- |
|  |  | Item 1 | Item 2 | Item 3 | Item 4 |  | Item 6 | Item 7 | Item 8 |  |  |
| Andersson | cohort | 1 | 1 | 1 | 0 | 2 | 1 | 1 | 1 | 8 | High |
| Arbel | cohort | 1 | 0 | 1 | 0 | 1 | 1 | 0 | 1 | 5 | moderate |
| Auvigne | cohort | 1 | 0 | 1 | 0 | 1 | 1 | 0 | 1 | 5 | moderate |
| Chae | cohort | 1 | 1 | 1 | 0 | 1 | 1 | 0 | 1 | 6 | moderate |
| Fabiani | cohort | 1 | 1 | 1 | 0 | 2 | 1 | 0 | 1 | 7 | High |
| Fabiani-XBB | cohort | 1 | 0 | 1 | 0 | 2 | 1 | 0 | 1 | 6 | moderate |
| Huiberts | cohort | 1 | 1 | 1 | 0 | 2 | 1 | 0 | 1 | 7 | High |
| Lin | cohort | 1 | 1 | 1 | 0 | 1 | 1 | 1 | 1 | 7 | High |
| Mateo-Urdiales | cohort | 1 | 1 | 1 | 0 | 2 | 1 | 1 | 1 | 8 | High |
| McConeghy | cohort | 0 | 1 | 1 | 0 | 1 | 1 | 1 | 1 | 6 | moderate |
| Mimura | cohort | 1 | 1 | 1 | 0 | 1 | 1 | 1 | 1 | 7 | High |
| Shrestha | cohort | 1 | 1 | 1 | 0 | 2 | 1 | 1 | 1 | 8 | High |
| Stecher | cohort | 1 | 1 | 1 | 0 | 2 | 1 | 1 | 1 | 8 | High |
| Tan | cohort | 1 | 1 | 1 | 0 | 1 | 1 | 1 | 1 | 7 | High |
| Tseng | cohort | 1 | 1 | 1 | 0 | 1 | 1 | 1 | 1 | 7 | High |
| Arashiro | Test-negative case-control | 0 | 1 | 0 | 0 | 1 | 0 | 1 | 0 | 3 | Low |
| Kirsebom | Test-negative case-control | 1 | 1 | 0 | 0 | 2 | 1 | 1 | 1 | 7 | High |
| Link Gelles | Test-negative case-control | 1 | 1 | 1 | 0 | 2 | 0 | 1 | 1 | 7 | High |
| Link-Gelles-XBB | Test-negative case-control | 0 | 1 | 1 | 0 | 2 | 0 | 1 | 1 | 6 | moderate |
| Surie | Test-negative case-control | 0 | 1 | 0 | 0 | 1 | 1 | 1 | 1 | 5 | moderate |
| Tartof | Test-negative case-control | 1 | 1 | 0 | 0 | 2 | 1 | 1 | 1 | 7 | High |
| Tenforde | Test-negative case-control | 0 | 1 | 0 | 0 | 2 | 1 | 1 | 1 | 6 | moderate |

Note: (1) Cohort studies: Item1:Representativeness of the exposure cohort.Item2:Selection of the non-exposure cohort. Item3:Determination of exposure.Item4:Diseases for which no study subjects had already developed the disease under study at the start of the study.Item5:Comparability of exposure and non-exposure cohorts.Item6:Methods of measurement of outcomes.Item7:Is the duration of follow-up sufficiently long for the disease under study? Item8:Completeness of follow-up. (2) Case-control studies: Item1:Is the definition of cases adequate? Item2:Representativeness of cases.Item3:Selection of controls. Item4:Definition of controls.Item5:Comparability of cases and controls based on design or analysis.Item6:Determination of exposures.Item7:Were the same methods used to determine exposures for cases and controls.Item8:Nonresponse rate. 0, 1, 2 means 0, 1, 2 points respectively.

**Table S2 Characteristics of the study**

| **Study** | **Year** | **Bivalent** | **Study Design** | **Age** | **Study Period** | **Population†** | **Country** | **Intervention group(bivalent vaccines)** | **Control groups(monovalent vaccines)** | **Epidemic strain(Omicron dominance period)** | **Outcome** | **Follow-up period** | **Relative vaccine effectiveness,%(rVE %, 95% CI)** |
| --- | --- | --- | --- | --- | --- | --- | --- | --- | --- | --- | --- | --- | --- |
| Andersson | 2023 | Ancestral + BA.4-5 or BA.1 | cohort | ≥50 | 2022.07-2023.04 | 3588054; 2676323 | Denmark, Finland, Norway and Sweden | 4 dose | 3 dose | BA.5、BQ、BF and XBB | Hospital admission | 8-90 d& | 1)BA.4-5 Bivalent vaccine: 67.8 (63.1 to 72.5)； 2)BA.1 Bivalent vaccine: 65.8 (59.1 to 72.4) |
|  |  |  |  |  |  |  |  |  |  |  | Death | 8-90 d& | 1)BA.4-5 Bivalent vaccine: 69.8 (52.8 to 86.8)； 2)BA.1 Bivalent vaccine: 70.0 (50.3 to 89.7) |
| Arashiro | 2023 | Ancestral + BA.4-5 or BA.1 | Test-negative case-control | ≥16 | 2022.09-2022.12 | 3498; 2693 | Japan | 3-5 dose | 3-4 dose | BA.5 | Symptomatic | ≥14 d&；14-90d # | 1 (−36 to 28) |
| Arbel | 2023 | Ancestral + BA.4-5 | retrospective cohort | ≥65 | 2022.09-2023.01 | 569519; 134215 | Palestine | 3-4 dose | NR | BA.5 and BQ.1 | Hospital admission | 68 d& | 72 (60 to 81) |
|  |  |  |  |  |  |  |  |  |  |  | Death | 68 d& | 68 (42 to 82) |
| Auvigne | 2023 | Ancestral + BA.4-5 | Matched cohort | ≥60 | 2022.10-2022.11 | 136852; 68426 | France | 3-4 dose | NR | BA.5 | Symptomatic | median 77d& | 8 (0 to 16) |
| Chae | 2023 | Ancestral + BA.4-5 or BA.1 | retrospective cohort | ≥18 | 2022.10-2022.12 | 58490; 29245 | South Korea | 1-2 booster dose | 1-2 booster dose | BA.5、BN.1 and XBB | Infection | 185 d& | 12.2 (6.5 to 17.7) |
| Fabiani | 2023 | Ancestral + BA.4-5 | retrospective cohort | ≥60 | 2022.09-2022.12 | 11190236; 1205353 | Italy | 2 booster dose | 1 booster dose | BA.5(B.1.1.529.5) (> 90%) | Severe COVID-19 | 7-90 d& | 58.7 (54.6 to 62.5) |
| Fabiani-XBB | 2023 | Ancestral + BA.4-5 | retrospective cohort | ≥60 | 2023.04-2023.06 | 11879461; 1970598 | Italy | 2-3 booster dose | 1 booster dose(99%) | XBB.1.5 and other XBB sublineages(88%) | Severe COVID-19 | 15-265 d&; 120 d# | 45.6(1.6 to 69.9) (15-60 d&) 24.7(10.5 to 36.7) (61-120 d&) 17.0(8.4 to 24.8) (121-180 d&) 14.3(1.6 to 25.3) (181-265 d&) |
| Huiberts | 2023 | Ancestral + BA.1 | prospective cohort | 18-85 | 2022.09-2022.12 | 32542; 17404 | Netherlands | ≥2 booster dose | 1 booster dose | BA.5(dominant) and BQ.1. | Infection | median 33-39 d& | 18-59 y 31 (18 to 42) 60-85 y 14 (3 to 24) |
| Kirsebom | 2023 | Ancestral + BA.1 | Test-negative case-control | ≥50 | 2022.09-2023.02 | 9954; 39108 | UK | 3 dose | ≥2 booster dose | BA.2,4-5(09/2022);BQ.1(10/2022);XBB.1.5（01/2023） | Hospital admission | 2-10 w&;＞6 m # | 53.0 (47.9 to 57.5) (2-4 w&) 46.3% (42.2 to 50.1) (5-9 w&) 35.9% (31.4 to 40.1) (≥10 w&) |
|  |  |  |  |  |  |  |  |  |  |  | Severe COVID-19 | 2-10 w&;＞6 m # | 58.5% (42.4 to 70.0) (2-4 w&) 52.0% (39.2 to 62.2) (5-9 w&) 48.3% (35.5 to 58.5) (≥10 w&) |
| Lin | 2023 | Ancestral + BA.4-5 | cohort | ≥12 | 2022.09-2022.12 | 6242259; 1070136 | USA | 1-3 booster dose | 1-2 booster dose | BA.4.6, BA.5, BQ.1和BQ.1.1 | Hospital admission | 15-99 d #& | 33.5 (2.9 to 62.1) |
|  |  |  |  |  |  |  |  |  |  |  | Severe COVID-19 | 15-99 d #& | 36.9 (12.6 to 64.3) |
| Link Gelles | 2022 | Ancestral + BA.4-5 | Test-negative case-control | ≥18 | 2022.09-2022.11 | 5800; 16474 | USA | 1 booster dose | 2-4 booster dose | BA.4/BA.5 | Symptomatic | 1 m &; ＞2 m # | 18-49y 30 (22 to 37) (2-3 m#) 43 (38 to 48) (4-5 m#) 46 (41 to 50) (6-7 m#) 56 (53 to 58) (≥8 m#) 50-64y 31 (24 to 38) (2-3 m#) 36 (38 to 41) (4-5 m#) 38 (32 to 43) (6-7 m#) 48 (45 to 51) (≥8 m#) ≥65y 28 (19 to 35) (2-3 m#) 33 (27 to 39) (4-5 m#) 36 (29 to 41) (6-7 m#) 43 (39 to 46) (≥8 m#) |
| Link-Gelles-XBB | 2023 | Ancestral + BA.4-5 | Test-negative case-control | ≥18 | 2022.12-2023.01 | 2969; 5289 | USA | 1 booster dose | 2-4 booster dose | BA.5、XBB/XBB.1.5(>50%) | Symptomatic | ≥2 w& | BA.5 18-49y: 52 (48 to 56)  50-64y: 43 (36 to 49) ≥65y: 37 (28 to 44)  XBB/XBB.1.5 18-49y: 49 (41 to 55) 50-64y: 40 (28 to 50) ≥65y: 43 (29 to 55) |
| Mateo-Urdiales | 2023 | Ancestral + BA.4-5 or BA.1 | retrospective matched cohort | ≥60 | 2022.09-2023.01 | 2129559; 1190756 | Italy | 2 booster dose | 2 booster dose | BA.5(>90%) | Severe COVID-19 | 14-118 d # | 60-79 y 53.6 (46.8 to 59.5) ≥80 y 48.3 (41.9 to 54.0) |
| McConeghy | 2022 | NR | cohort | ≥50 | 2022.03-2022.06 | 3804; 1902 | USA | 2 booster dose | 1 booster dose | BA.2 and BA.2.12.1(2022.3-6); BA.4/5(2022.7) | Infection | 0-60 d & | 25.8(1.2 to 44.3) |
|  |  |  |  |  |  |  |  |  |  |  | Hospital admission | 0-60 d & | 60.1(−18.8 to 91.5) |
|  |  |  |  |  |  |  |  |  |  |  | Death | 0-60 d & | 89.6(45.0 to 100.0) |
|  |  |  |  |  |  |  |  |  |  |  | Severe COVID-19 | 0-60 d & | 73.9(36.1 to 92.2) |
| Mimura | 2023 | Ancestral + BA.4-5 or BA.1 | population-based cohort | ≥65 | 2019.01-2023.02 | 81977; 57396 | Japan | 2-3 booster dose | 1-2 booster dose | Ba.5(2022.7-12) | Infection | ≥90 d #; 0-13 d&/≥14 d& | 43.5 (36.3-49.9)(0-13 d&) 57.9 (52.7-62.5)(≥14 d&) |
|  |  |  |  |  |  |  |  |  |  |  | Hospital admission | ≥90 d #; 0-13 d&/≥14 d& | 70.9(19.0-89.6)(0-13 d&) 77.5(46.7-90.5)(≥14 d&) |
|  |  |  |  |  |  |  |  |  |  |  | Death | ≥90 d #; 0-13 d&/≥14 d& | 83.3(29.8-96.0)(0-13 d&) 77.3(44.3-90.7)(≥14 d&) |
| Shrestha | 2023 | Ancestral + BA.4-5 | cohort | ≥18 | 2022.09-2023.02 | 48141; 12789 | USA | ≥1 booster dose | ≥1 booster dose | BA.4/5、BQ、XBB | Cumulative incidence | mean(SD), 263(142) d # | BA.4/5, 29 (21 to 37) BQ dominant phase,20 (6 to 31) XBB dominant phase,4 (-12 to 18) |
| Stecher | 2023 | Ancestral + BA.4-5 or BA.1 | cohort | ≥75 | 2022.07-2023.01 | 408073; 60403 | Norway | 4 dose | 3 dose | NR | Death | ≥24 w #; 2-9 w & | BA.1 Bivalent vaccine: 67 (60 to 72) BA.4-5 Bivalent vaccine: 60 (52 to 67) |
| Surie | 2022 | Ancestral + BA.4-5 | Test-negative case-control | ≥65 | 2022.09-2022.11 | 20; 59 | USA | 3 booster dose | ≥2 booster dose | BA.5/BQ.1/BQ.1.1 | Hospital admission | ≥2 m # | 73 (52 to 85) |
| Tan | 2023 | Ancestral + BA.4-5 or BA.1 | retrospective cohort | ≥18 | 2022.10-2023.01 | 2749819 | Singapore | 4 dose | 4 dose | XBB | Hospital admission | ≥5 m # | 90 (85 to 93) |
|  |  |  |  |  |  |  |  |  |  |  | Symptomatic | ≥5 m # | 83 (82 to 84) |
| Tartof | 2023 | Ancestral + BA.4-5 | Test-negative case-control | ≥18 | 2022.08-2023.04 | 24246; 99173 | USA | ≥3 dose | ≥2 booster dose | BA.4/5(2022.8-2023.1); XBB(2023.1.22-) | Critical illness | 28-112 m #; ≥8 w & | 50(23 to 68) |
|  |  |  |  |  |  |  |  |  |  |  | Hospital admission | 28-112 m #; ≥8 w & | 39(28 to 49) |
| Tenforde | 2023 | Ancestral + BA.4-5 | Test-negative case-control | ≥18 | 2022.09-2022.11 | 3658; 247 | USA | ≥1 booster dose | 2-4 dose | BA.5 | ED/UC encounters | ≥2 m #; ≥7 d & | 32 (21-42) (2-4 m#) 43 (34-50) (5-7 m#) 54 (48-59) (8-10 m#) 50 (44-56) (≥11 m#) |
|  |  |  |  |  |  |  |  |  |  |  | Hospital admission | ≥2 m #; ≥7 d & | 42 (20-58) (5-7 m#) 44 (23-59) (8-10 m#) 48 (30-62) (≥11 m#) |
| Tseng | 2023 | Ancestral + BA.4-5 | matched cohort | ≥6 | 2022.08-2023.01 | 341265; 290292 | USA | ≥1 booster dose | ≥2 dose | BA.5、BQ.1、XBB | Hospital admission | ≥8 w #; 0-3 m& | 70.3 (64.0 to 75.4) |
|  |  |  |  |  |  |  |  |  |  |  | Death | ≥8 w #; 0-3 m& | 82.7 (63.7 to 91.7) |
|  |  |  |  |  |  |  |  |  |  |  | Infection | ≥8 w #; 0-3 m& | 35.9 (32.7 to 39.0) |

Note: & Clinical outcome monitoring intervals since booster dose of bivalent COVID-19 vaccine; # Clinical outcome monitoring intervals since dose of monovalent COVID-19 vaccine (d: days; w: weeks; m: months); year,y; confidence interval, CI.

†Cohort study, total number of participants; number of bivalent vaccine participants. Case-control study, cases; controls

**A B**

Figure S1 Risk of publication bias assessment of the relative effectiveness of bivalent vaccines.

1. COVID-19-associated infections or Symptomatic Infection. Egger’s test: Test result: t = -1.65, observed = 10, imputed = 2, p-value = 0.1370, Using the trim-and-fill method to address publication bias, the adjust rVE% was 36.94% (95%CI, 16.95 to 56.94). B) COVID-19-associated hospitalization. Egger’s test: Test result: t = -0.36, p-value = 0.7183.

‘

A B

Figure S2 Sensitivity analysis of the relative effectiveness of bivalent vaccines

A.COVID-19-associated infections or Symptomatic Infection. B.COVID-19-associated hospitalization.
